# Tuneable Permeability of Cellulose Nanofibrils‐based Membranes in Next‐Generation Barrier‐On‐Chip Systems

**DOI:** 10.1002/cbic.202500843

**Published:** 2026-04-04

**Authors:** Vita Guarino, Johan Erlandsson, Elisa De Luca, Elisabetta Perrone, Alessandra Zizzari, Monica Bianco, Albert Portone, Stefano Leporatti, Lars Wågberg, Giuseppe Gigli, Lorenzo Moroni, Valentina Arima

**Affiliations:** ^1^ University of Salento Department of Experimental Medicine c/o Campus Ecotekne Lecce Italy; ^2^ Tecnomed Puglia ‐ Tecnopolo per la medicina di precisione (Biotech Lecce Hub) c/o Campus Ecotekne Lecce Italy; ^3^ Institute of Nanotechnology of Consiglio Nazionale delle Ricerche (CNR NANOTEC) c/o Campus Ecotekne Lecce Italy; ^4^ Division of Fibre Technology Department of Fibre and Polymer Technology School of Chemistry Biotechnology and Health KTH Royal Institute of Technology Stockholm Sweden; ^5^ Center for Biomolecular Nanotechnologies (CBN) of Istituto Italiano di Tecnologia (IIT) Arnesano Italy; ^6^ Maastricht University department of complex tissue regeneration MERLN Institute for Technology‐Inspired Regenerative Medicine Maastricht Netherlands

**Keywords:** cellulose, cellulose nanofibrils, endothelial cells, In vitro model, organ‐on‐chip

## Abstract

Barriers in the human body play a crucial role in regulating the exchange of substances between compartments, with permeability alterations occurring under both physiological and pathological conditions. In vitro barrier models are essential tools for studying the mechanisms of molecular diffusion across these barriers. Traditional coculture systems or advanced organ‐on‐chip (OoC) platforms mostly utilize permeable membranes based on artificial, nonbiodegradable materials. In this study, we introduced cellulose nanofibrils (CNFs)‐based membranes to develop a new class of in vitro barrier systems. CNFs, derived from natural sources, are nontoxic, biodegradable, optically transparent, and feature a 3D fibrillar structure that mimics the cellular basement membrane. We successfully modulated the permeability of CNF‐based membranes, interposed in dual‐chamber polydimethylsiloxane devices, to small molecules through chemical and enzymatic treatments, while preserving their ability to allow cell adhesion and growth. This technology holds potential for its integration in next‐generation OoC devices, offering more realistic and complex models that closely mimic the physiological behavior of human barriers.

## Graphical abstract

Cellulose nanofibril (CNF)‐based membranes enable tuneable permeability in dual‐chamber PDMS barrier models. Their fibrillar 3D architecture closely mimics the native basement membrane, supporting cell adhesion and growth while allowing controlled modulation of small‐molecule diffusion through chemical and enzymatic treatments—a biomimetic platform for next‐generation organ‐on‐chip systems.

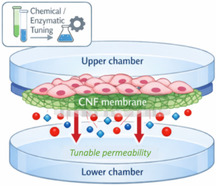

## 1 Introduction

Within the human body, barriers play a vital role in protecting against harmful substances and regulating the exchange of materials [1]. Understanding the mechanisms behind these barrier tissues is essential for developing effective pharmaceutical treatments [2, 3]. Traditional cellular coculture systems and advanced Organ‐on‐Chip (OoC) platforms have been employed to create in vitro models of these barriers. These models are invaluable tools for determining how substances cross specific cellular barriers and for assessing their impact on barrier integrity [1, 3].

Both traditional coculture and OoC systems depend on semipermeable artificial membranes of polycarbonate (PC), polyethylene terephthalate (PET), or polydimethylsiloxane (PDMS) to create distinct microenvironments that support cell growth and differentiation. The pore structure of commercial track‐etched membranes significantly influences fluid dynamics and solute permeability, allowing for communication between cell layers [4]. However, simply compartmentalizing cells using artificial porous membranes does not fully replicate the complexity of the native basement membrane (BM) found in tissues. The BM is a specialized extracellular matrix (ECM) that anchors to cell surfaces, showing a 3D branching network of fine filaments and cords [5] and it is crucial for inducing physiological cell phenotypes and providing an interactive interface between cell and surrounding environment that can act as a selective barrier within and between these compartments [6, 7].

Structurally, the BM is arranged as a thin, sheet‐like matrix whose thickness and composition vary according to the tissue of origin, composed of a variety of proteins, including collagen, fibronectin, and laminin, organized into well‐defined nano‐ to microscale fibrillar structures. This hierarchical organization endows the BM with unique physical and mechanical properties that are essential for regulating cell behavior [8].

Due to its intrinsic complexity and variability, faithfully recapitulating the biochemical and structural cues of the native BM in vitro remains a significant challenge. Although porous flat membranes—such as those made of PDMS, PC, or PET—are widely used because they are relatively simple and cost‐effective to fabricate via soft lithography or track‐etching techniques, they present several critical limitations. In particular, these membranes lack the fibrillar architecture that is a hallmark of the in vivo BM. Moreover, their fabrication processes are generally incompatible with the incorporation of BM proteins, resulting in the absence of key biochemical cues [7].

Additional drawbacks are associated with the intrinsic material properties of these membranes. Silicone‐based membranes, such as PDMS, are highly hydrophobic and prone to the nonspecific absorption of drugs and biomolecules [9, 10]. In contrast, PC and PET membranes typically exhibit high stiffness and thickness, which represent major challenges for the development of BM‐mimicking structures [4]. These factors also affect membrane permeability, a crucial parameter for enabling molecular transport, cell–cell communication, and proper cellular function.

Therefore, the limitations of artificial porous membranes to emulate the intricate cellular interactions and biophysical properties of the native BM underscores the need for biomimetic membranes that better replicate the natural environment of cellular barriers.

Recent advances in OoC systems have demonstrated great potential for creating more biomimetic in vitro barrier models through the integration of *ad hoc* engineered membranes [7]. One promising method involved incorporating electrospun membranes into OoC devices. These membranes, made of randomly stacked micro‐ or nanofibers, were able to mimic the 3D porous structure of the native BM. Materials like poly(lactic‐co‐glycolic acid), polycaprolactone, gelatine, and poly(methyl methacrylate) have been used to fabricate electrospun constructs that support cell viability and growth [11, 12]. Additionally, collagen‐based enzymatically degradable membranes resulted in a biologically relevant alternative, without the need for electrospinning, mimicking BM remodeling and allowing for controlled permeability [13]. Finally, previous studies have introduced ‘paper‐fluidics’ systems, which integrate scaffold‐based and microfluidic strategies [14, 15, 16, 17]. In these platforms, paper‐ or cellulose‐based membranes act as both structural barriers separating fluidic microchannels or microcompartments and as biologically compatible scaffolds that support cell culture, allow nutrient exchange, and support mechanical stimulation. By leveraging the intrinsic porosity, flexibility, and ease of handling of such membranes, ‘paper‐fluidics’ offers a low‐cost, scalable approach to creating functional in vitro models, particularly suitable for tissue barrier studies.

For instance, Duong et al. developed cellulose‐based membranes integrated into microfluidic platforms for blood–brain barrier (BBB) modeling [18]. Their dual‐layer PDMS device incorporated a filter paper membrane, serving simultaneously as a physical barrier and a 3D scaffold for cell attachment and mechanical stimulation. Despite these advantages, the system exhibited notable limitations, including intrinsic autofluorescence that hindered fluorescence‐based imaging, optical opacity caused by densely packed cellulose fibers, and restricted porosity that limited direct cell–cell interactions [17, 18].

In this study, we investigated films of carboxymethylated cellulose nanofibrils (CNFs), elsewhere referred to as ‘nanopapers’, as alternatives to conventional porous membranes of in vitro barrier systems. Moreover, CNFs films are a superior alternative to conventional cellulose paper, given its enhanced mechanical strength, transparency, biodegradability, and tuneable properties [7, 19, 20, 21]. CNFs films consist of randomly oriented fibrils organized into dense 3D networks [22], resembling the fibrous architecture of the in vivo BM, with the added advantage of being more cost‐effective than ECM‐derived materials like collagen [19]. Past research by Skogberg et al. (2017) already demonstrated that CNFs films, produced via droplet casting, can serve as effective cell culture substrates, also promoting cell orientation on aligned cationic CNFs surfaces [23]. Despite these promising attributes, CNFs films have been utilized primarily in energy storage, electronics, water treatment, and high‐performance packaging [22].

This study exploits transparent films of pure carboxymethylated CNFs, produced via dead‐end filtration [24], as membranes incorporated into PDMS dual‐chamber devices for in vitro barrier modeling applications. We propose that CNF‐based membranes can be structurally and chemically tuned to achieve controlled permeability—via both chemical and enzymatic treatments—thereby enabling precise customization for specific experimental needs. We performed detailed analyses of untreated/treated CNFs membrane morphology, stiffness and structure and assessed their suitability for culturing endothelial cells. Overall, our findings broaden the potential biomedical applications of CNFs‐based materials.

## 2 Results

### 2.1 Integration of CNF‐based Membranes into PDMS Devices for Cell Culture

**FIGURE 1:**
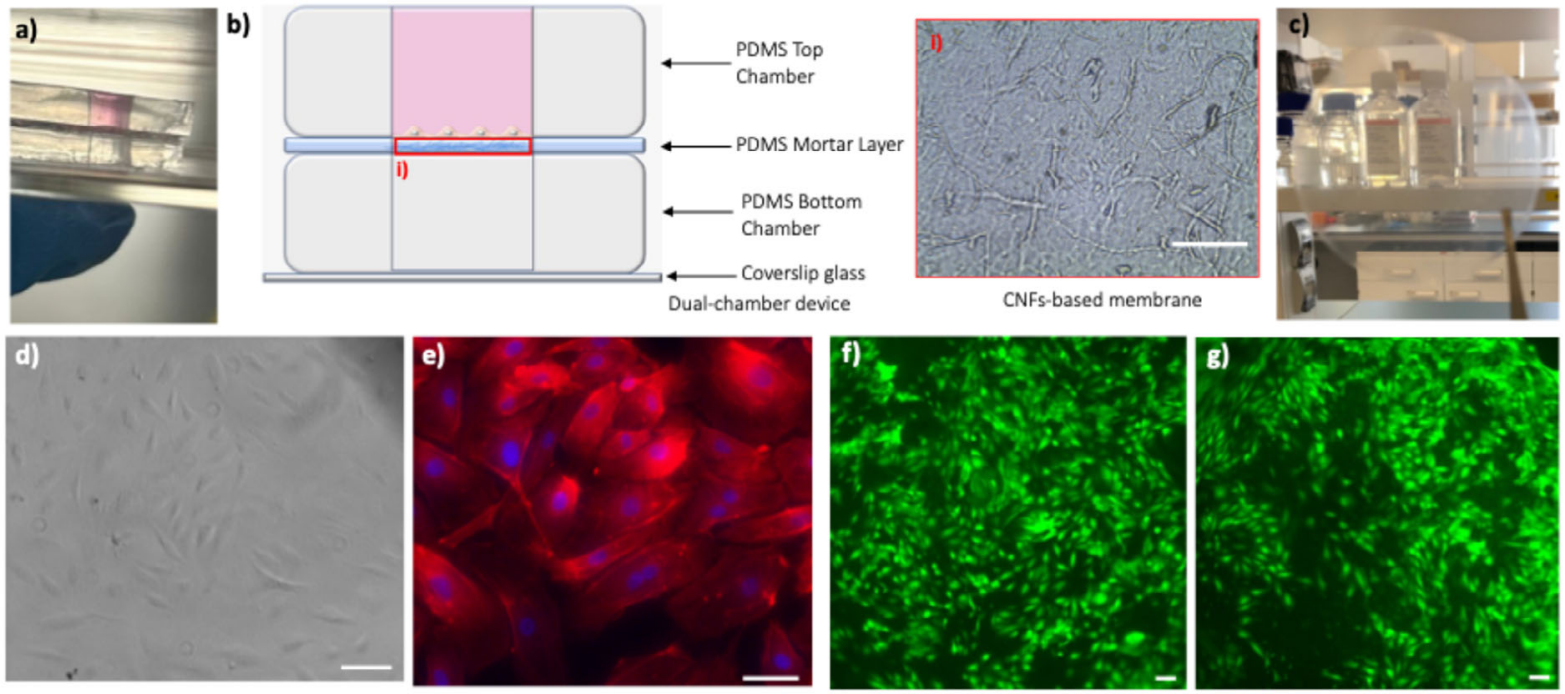
Dual‐chamber device incorporating a CNF‐based membrane for cell culture. (a) Photograph of the assembled dual‐chamber device filled with cell culture medium and placed in a Petri dish. (b) Schematic representation of the device: the inner faces of the PDMS layers, each with a central milli‐chamber, are coated with a thin PDMS mortar layer to bond the interposed CNF‐based membrane ((i) bright‐field image with scale bar of 100 µm). (c) Photograph of the highly transparent CNF‐based membrane prior to device integration. (d) Bright‐field image of viable HCMEC/D3 cells on an untreated CNF‐based membrane (scale bar: 100 µm). (e) Fluorescence image of fixed HCMEC/D3 cells stained for actin filaments (red) and counterstained with DAPI for nuclei (blue) on an untreated CNF‐based membrane (scale bar: 50 µm). (f) Live‐cell imaging of calcein‐stained HCMEC/D3 cells on a cellulase‐treated CNF‐based membrane (scale bar: 100 µm). (g) Live‐cell imaging of calcein‐stained HCMEC/D3 cells on a NaOH‐treated CNF‐based membrane (scale bar: 100 µm). All experiments were performed in triplicate (*n* = 3).

To compare ‘nanopaper’‐based devices with traditional ‘paper‐fluidics’, immortalized human cerebral microvascular endothelial cells (HCMEC/D3)—a widely used in vitro BBB model [25, 26]—were cultured under static conditions in a dual‐chamber device (Figure 1a,b), with the CNF‐based membrane positioned between compartments using a straightforward PDMS mortar assembly (inset of Figure 1b). Because device assembly was performed manually, a limited number of samples may exhibit slight misalignment (shift ≥500 μm), as shown in Figure 1a; however, this does not affect cytocompatibility assessments. The high transparency of the CNF‐based membrane allowed clear bright‐field visualization of cell adhesion and confluency (Figure 1c,d), also confirmed by fluorescent imaging after fixing and staining (Figure 1e–g).

We explored two approaches to modulate membrane permeability, as fully described in the next section, while preserving its integrity and transparency: (i) chemical modification, by applying a 2 wt% NaOH solution to the upper compartment for 2 min at room temperature followed by triple rinsing with distilled water (higher concentrations or longer exposure times led to degradation), and (ii) enzymatic modification, by incubating membranes with 5 mg/mL cellulase from *Trichoderma* sp. in phosphate‐buffered saline (PBS, pH 7.0) for 3 h at 37°C followed by thorough washing (prolonged exposure >24 h or higher enzyme concentrations resulted in dissolution).

CNF‐based membranes cytocompatibility was confirmed by calcein‐staining of HCMEC/D3 grown into the dual‐chamber device after both NaOH and cellulase treatments (Figure 1f,g). Compared to standard cellulose fibers, CNF‐based membranes display optical transparency and negligible autofluorescence due to their nanoscale dimensions—which minimize light scattering—and their high chemical purity resulting from delignification and bleaching, processes that remove lignin and other chromophoric impurities. In contrast, conventional cellulose fibers retain microscale structures and residual lignin‐ or carbonyl‐based chromophores, which lead to opacity and intrinsic autofluorescence [27, 28, 29]. This property is inherent to the material and does not depend on the NaOH or cellulase treatments employed in our work to modulate membrane permeability; these treatments do not alter the native optical characteristics of CNFs.

Overall, our findings indicate that CNF‐based membranes combine high optical clarity, low autofluorescence, tuneable permeability, and high cytocompatibility, even after mild chemical or enzymatic modification. By addressing the key limitations of conventional cellulose‐based membranes, CNF‐based membranes offer a robust platform for real‐time, advanced imaging of barrier models. Beyond their use with static culture systems, these membranes can be seamlessly integrated into microfluidic platforms, offering a versatile substrate for building dynamic and physiologically relevant OoC models. Future work will aim to exploit their tuneable architecture to recreate different tissue‐specific barriers, expanding their applicability to a broader range of in vitro models.

### 2.2 Permeability Control of CNF‐based Membranes

CNF‐based membranes, produced via dead‐end filtration of carboxymethylated CNFs, possess nanometer‐scale pores and are renowned for their exceptional wet strength [24, 30]. Their porous architecture enables them to act as highly efficient nanosieves, effectively blocking the passage of macromolecules such as polyethylene glycols (PEGs, ≥4 kDa) [31]. Moreover, the nanoscale pore network supports ion selectivity, expanding their functional scope for separation and sensing applications [32].

To modulate transport properties and make them more suitable to in vitro barrier modeling, CNF‐based membranes integrated into our PDMS‐based dual‐chamber device were subjected to either chemical (NaOH) or enzymatic (cellulase) treatments. Untreated membranes, exposed only to water, served as controls. Rhodamine B (RhodB), a small fluorescent tracer (∼479 Da), was selected for the permeability assays due to its well‐characterized optical properties, high detection sensitivity, and suitability for real‐time diffusion studies [33]. Using a low molecular weight tracer in these preliminary diffusion tests allowed us to evaluate baseline permeability and structural integrity under controlled conditions.

**FIGURE 2:**
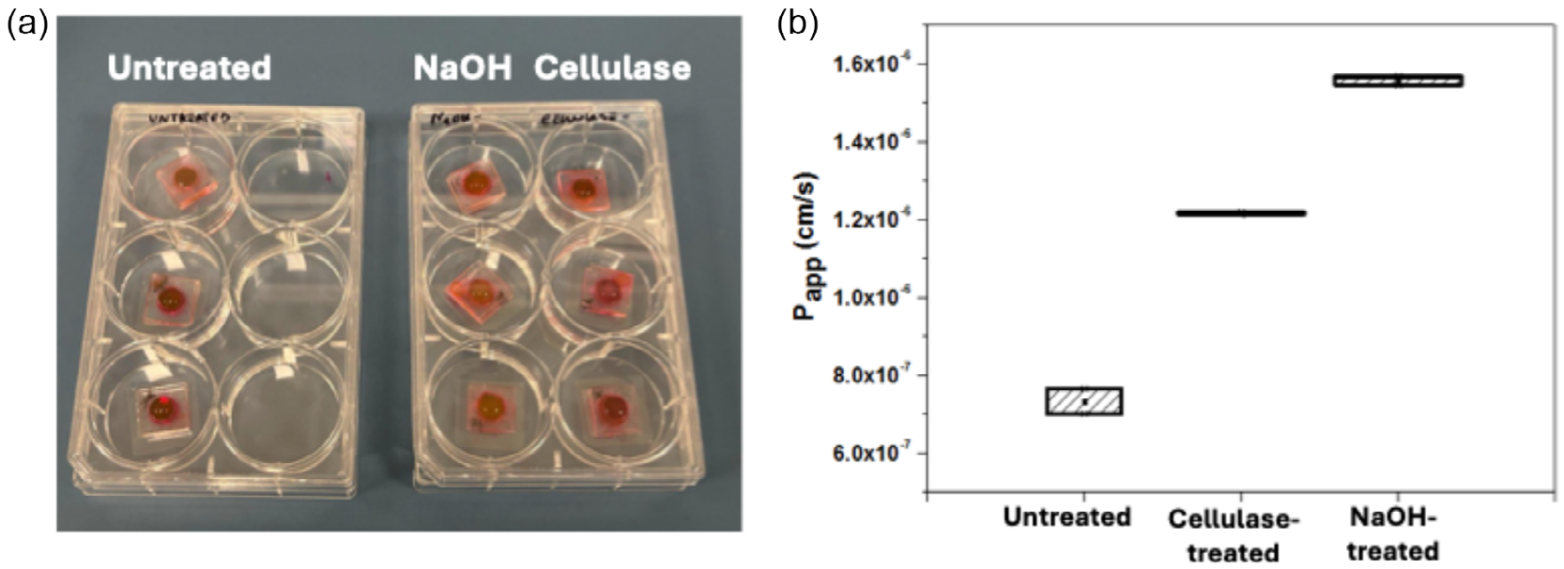
Permeability test of CNF‐based membranes to RhodB. (a) Photograph of dual‐chamber devices during the diffusion assay, with RhodB solution loaded in the upper chambers and distilled water in the lower chambers, performed in triplicate for each condition. The visual arrangement highlights the comparative setup for untreated, cellulase‐treated, and NaOH‐treated membranes. The devices are incubated in standard 6‐well plates. (b) Quantification of *P*_app_ to RhodB, showing a significant increase in permeability after both cellulase and NaOH treatments compared to untreated membranes, with NaOH yielding the highest enhancement. All experiments were performed in triplicate (*n* = 3). In Figure 2b, the mean value is indicated by the central spot within the box, while the box represents the variability expressed as the mean ± standard deviation.

A RhodB aqueous solution was introduced into the top chamber of the devices, while the bottom chamber was filled with distilled water (dH_2_O) (Figure 2a). After overnight incubation at 37°C, the absorbance of RhodB in the bottom chamber was measured to evaluate dye diffusion across the membrane. The apparent permeability (*P*_app_) values obtained were (0.732 ± 0.046) × 10^−6^ cm·s^−1^ for untreated membranes, (1.217 ± 0.003) × 10^−6^ cm·s^−1^ for cellulase‐treated membranes, and (1.560 ± 0.013) × 10^−6^ cm·s^−1^ for NaOH‐treated membranes (Figure 2b). Both chemical and enzymatic treatments significantly increased membrane permeability compared to the untreated condition, with NaOH treatment resulting in the highest enhancement.

The effect of NaOH treatment on permeability aligns with previous reports describing cellulose swelling and dissolution under alkaline conditions, depending on process parameters and presence of additives [34]. Dissolution involves the disintegration of cellulose fibers into rod‐like fragments, whereas swelling results from the conversion of the membrane from its proton (H^+^)‐form to its sodium (Na^+^)‐form. At high alkali levels, the dissociation of cellulose carboxyl groups (COOH) into carboxylates (COO^−^) creates an osmotic pressure gradient that drives water uptake into the fibrillar network to dilute ionic species [35]. This structural alteration likely increases the effective pore size, facilitating the diffusion of small molecules such as RhodB.

Similarly, enzymatic treatment with cellulase led to a marked increase in permeability. CNF‐based membranes are primarily composed of glucose units linked by β‐1,4 glycosidic bonds, which are specifically cleaved by cellulolytic enzymes into glucose monomers [36]. This enzymatic action partially disrupts the fibrillar network, enlarging pathways for molecular transport.

In summary, both NaOH and cellulase treatments provide effective routes to finely tune the permeability of CNF‐based membranes, enabling adjustment of their barrier properties without compromising mechanical integrity. This tunability is particularly valuable for in vitro barrier models, where matching physiological transport rates is essential. Building upon these findings, we next examined whether these treatments affect the membrane's capacity to absorb and subsequently release small molecules, an important factor in predicting their performance in dynamic biological systems.

### 2.3 Absorption and Release Ability of CNF‐Based Membranes

In addition to standard diffusion assays, we performed release experiments to investigate the interaction between RhodB and CNF‐based membranes. These tests aimed to assess the potential for nonspecific retention or binding of small molecules within the membrane matrix, a key factor in barrier model applications [37]. This strategy enabled the evaluation of both the release kinetics and the retention capacity of CNF‐based membranes under three different conditions (untreated, cellulase‐treated, or NaOH‐treated).

The same membrane samples previously used in the permeability experiments were sequentially incubated at 37°C in fresh PBS for three consecutive 24‐h cycles, mimicking the periodic medium changes typical of cell culture experiments. After each incubation, the collected PBS was analyzed for RhodB absorbance, which is directly proportional to the concentration of RhodB released from the membranes.

**FIGURE 3:**
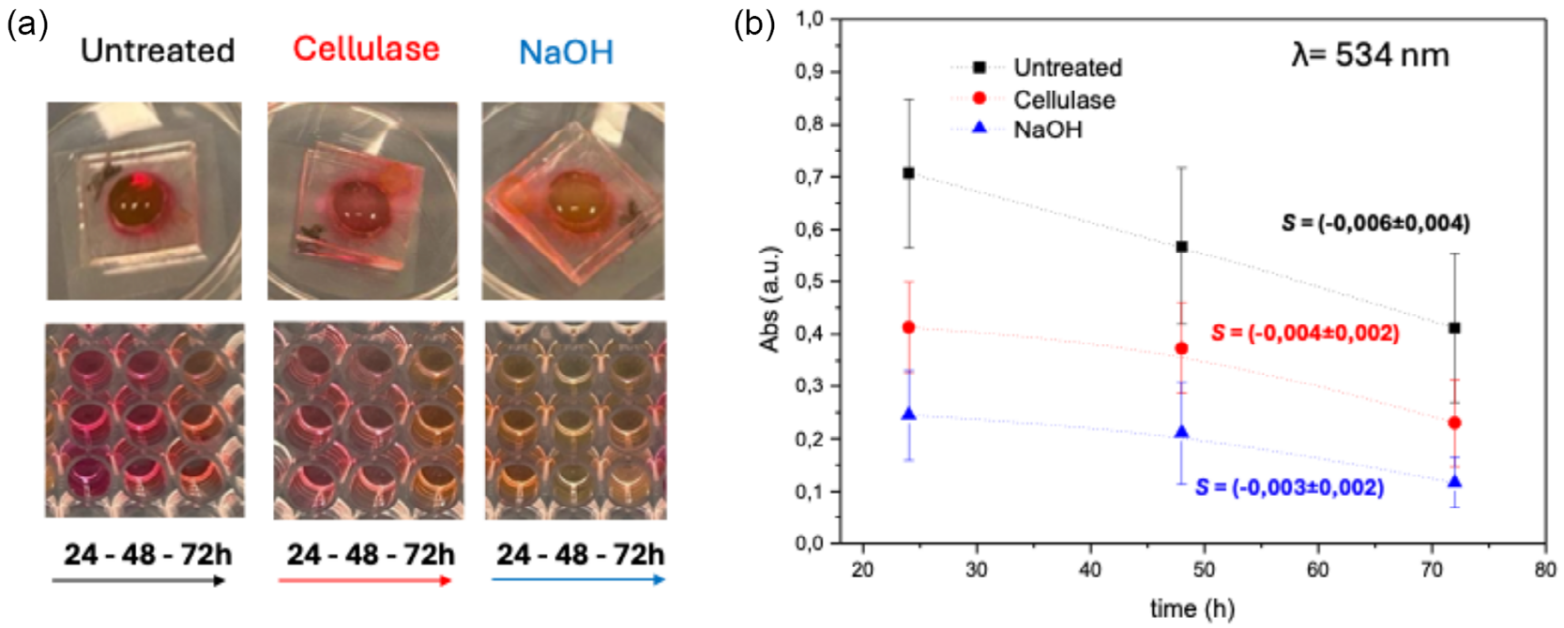
RhodB release from CNF‐based membranes (a) Upper part: images of untreated and cellulase‐ and NaOH‐treated CNF‐based membranes during PBS incubation for the release test. Lower part: images of solutions collected after membrane incubation in PBS for consecutively three cycles of 24 h. The experiments were repeated three times using three different RhodB‐loaded CNF‐based membranes for each condition. Therefore, aliquots from three different samples are shown. (b) Plot representing an average of the absorbance values (at λ = 534 nm) of the three RhodB solutions shown in the lower part of figure (a) to evaluate the release of RhodB over time. All experiments were performed in triplicate (*n* = 3). The error bars in Figure 3b are reported as mean ± standard deviation.

It should be noted that the samples shown in Figure 3a were not initially loaded with identical RhodB concentrations, as they were derived from the permeability experiments described in Section 2. For this reason, it was not possible to normalize the data to the initial loaded concentration, and a precise quantitative determination of release kinetics could not be performed. Nevertheless, qualitative comparisons can still provide meaningful insights.

Visually, untreated CNF‐based membranes appeared more intensely colored than treated ones, likely reflecting a higher initial RhodB loading. This observation is consistent with the permeability results: treated membranes exhibited greater RhodB transport from upper chamber to the lower chamber (higher permeability) and thus retained less dye. Absorbance analysis of the three PBS aliquots collected after 24, 48, and 72 h revealed a progressive release of RhodB over time, with an approximately linear trend (Figure 3b). While the slope appeared steeper for untreated membranes and gentler for treated ones, statistical analysis of the slopes (S) from linear fits, including error estimates, indicated that the release rate remained relatively constant across all samples over the 72‐h interval.

Overall, cellulase and NaOH treatments did not significantly affect the ability of CNF‐based membranes to release small molecules at regular 24‐h intervals. The release profiles confirmed that RhodB is not irreversibly adsorbed and can be effectively eluted. This property is particularly relevant for in vitro barrier models, as it ensures that soluble compounds—such as nutrients, metabolites, or pharmacological agents—can freely diffuse or be cleared during perfusion experiments, without significant loss due to membrane sequestration. Such behavior supports the suitability of CNF‐based membranes for long‐term dynamic culture systems, where controlled and predictable solute exchange between compartments is essential for physiological relevance.

### 2.4 Morphology Characterization of CNF‐Based Membranes

**FIGURE 4:**
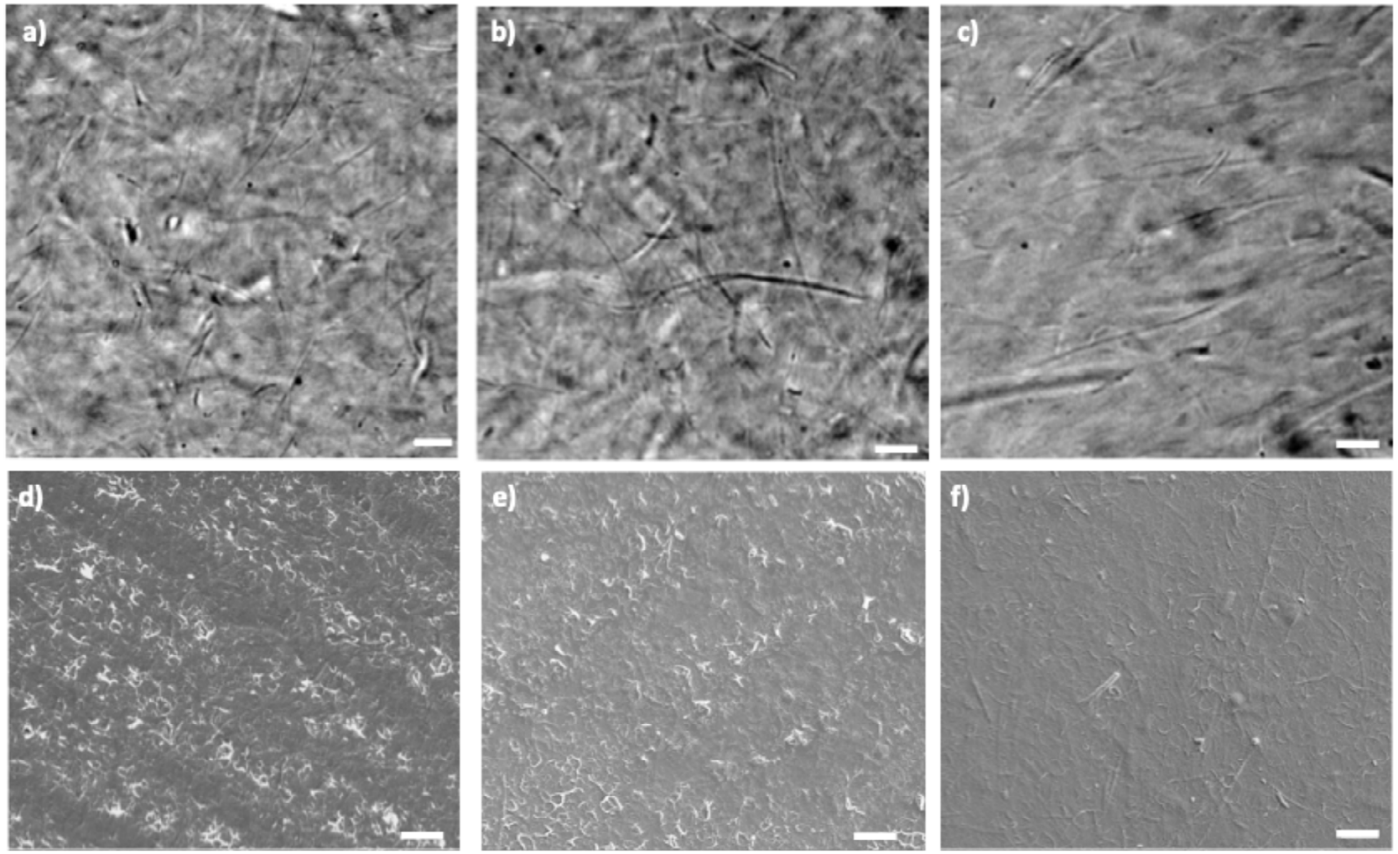
Morphology of CNF‐based membranes. (a–c) BF images of untreated, cellulase, and NaOH‐treated membranes (scale bar: 5 µm). (d–f) SEM images of untreated and cellulase‐ and NaOH‐treated membranes (scale bar: 10 µm). The BF and SEM images are obtained from the same samples.

For studying how NaOH and cellulase treatments affected the CNFs structure, the morphology of the membranes before and after the treatments was analyzed by bright‐field (BF) microscopy, scanning electron microscopy (SEM), and atomic force microscopy (AFM). Observations of BF (Figure 4a–c) and SEM images (Figure 4d–f) revealed that NaOH treatment resulted in a noticeable surface smoothing of the membranes, whereas untreated and cellulase‐treated membranes displayed highly similar morphologies, with no significant alterations at the microscale.

**FIGURE 5:**
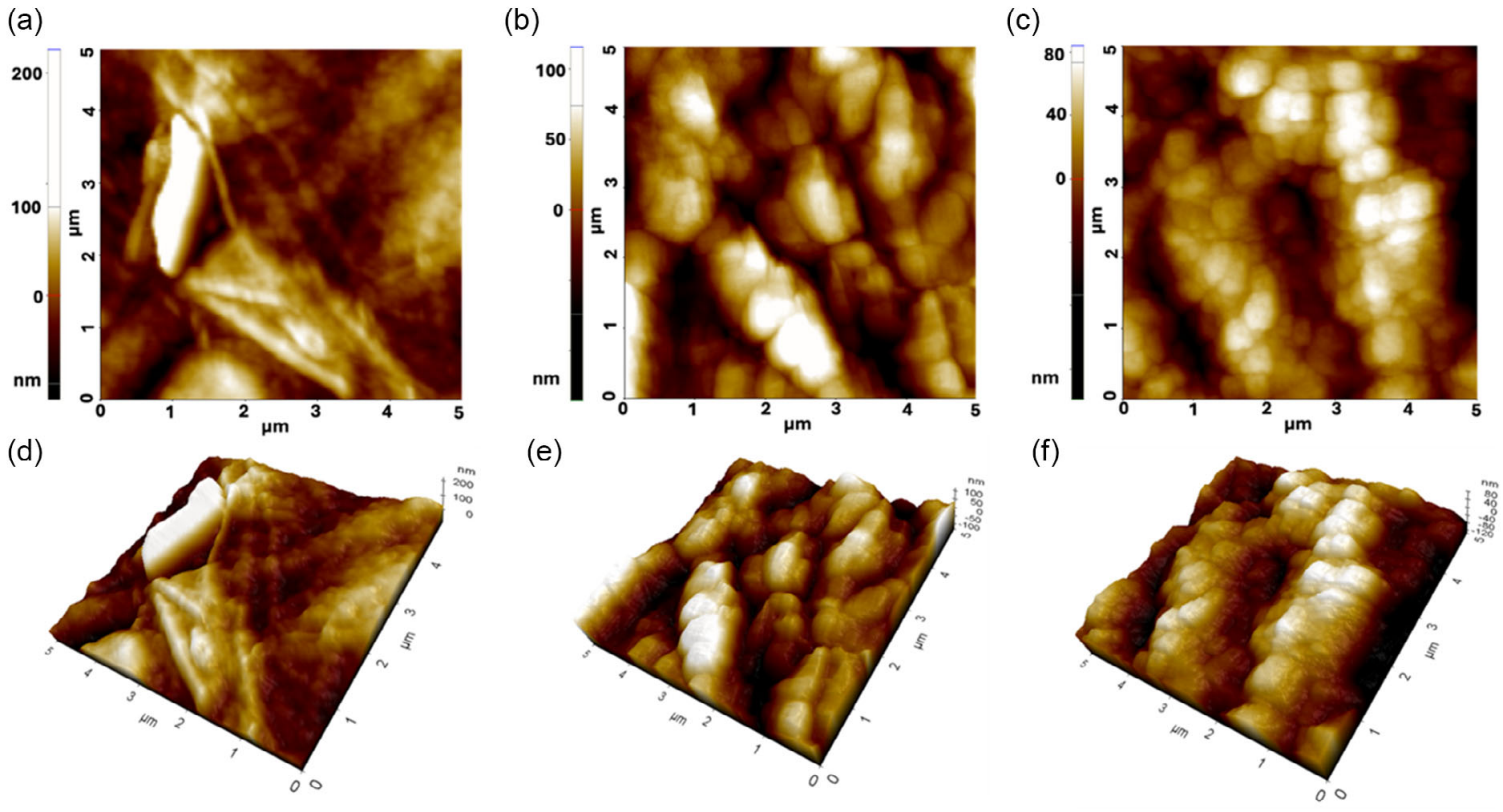
AFM images and corresponding 3D elaborations of (a,d) untreated CNF‐based membrane, (b,e) membrane treated with cellulase, and (c,f) membrane treated with NaOH.

However, AFM provided a higher level of structural detail, enabling visualization of nanoscale changes within the fibrillar network. AFM images of pure CNF‐based membranes at the scale of 5 µm x 5 µm showed the presence of fibers of different lengths and thicknesses (see Figure 5a and 3D image of Figure 5d) oriented in different directions on the substrate. Following treatment with cellulase, the membrane surface became less fibrous with a rearrangement into grains (Figure 5b and 3D image of Figure 5e). A similar granular organization was observed after NaOH treatment as shown in Figure 5c,f. The grain appearance due to fibrils modifications did not have an excessive impact on the root mean square roughness Rq (resulting in (39.2 ± 10.8) nm for untreated, (46.6 ± 2.9) nm and (42.5 ± 6.3) nm for cellulase‐ and NaOH‐treated CNFs membranes, respectively). It is important to point out that AFM images were acquired in the dried state after treatments; therefore, no changes in morphology and surface roughness due to fiber swelling can be observed.

**FIGURE 6:**
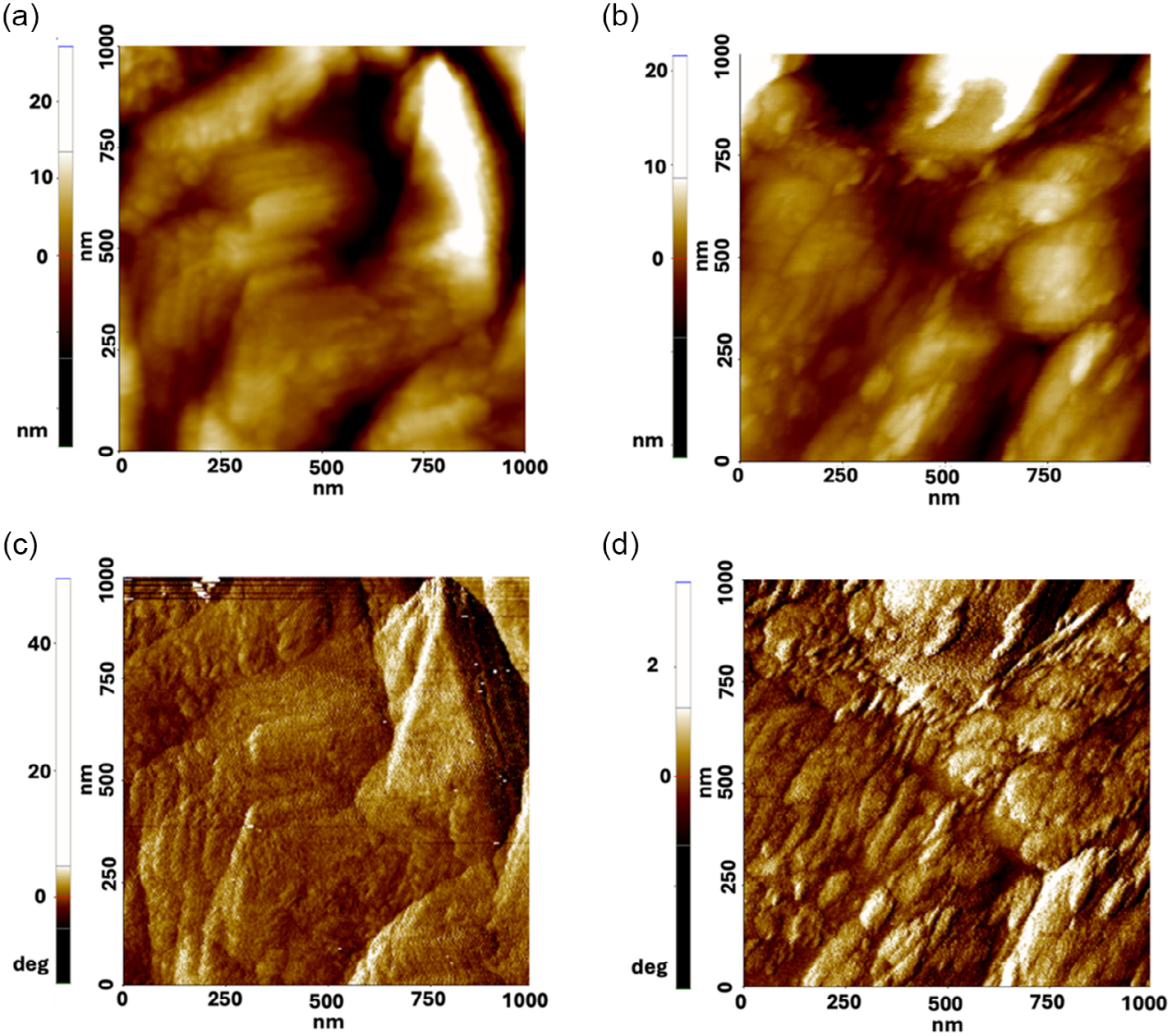
AFM images and corresponding phase images of (a,c) membrane treated with cellulase and (b,d) membrane treated with NaOH.

Both treatments led to modifications of morphology of the fibers and a partial loss of spatial three‐dimensionality of the structures. However, a difference between the two treatments was visible by investigating the structures of each grain. Images at 1 µm x 1 µm scale showed that grains originating from the cellulose treatment consisted of smaller nanofibers self‐assembled into compact grains (Figure 6a). Phase contrast images (Figure 6c) also confirmed this organization. On the other hand, a fragmentation into self‐organized nanofibers was not evident in AFM and phase contrast images of membranes treated with NaOH at higher magnification (Figure 6b,d), where each grain appeared to be formed by small rod‐like clusters of cellulose [38]. These results are considered compatible with the faster, less selective, and more disruptive chemical treatment with NaOH and with the more controlled mechanism of action of cellulase on the membrane surface along the direction of the fibrils.

In summary, both treatments transformed the morphology of CNFs fibers into granular structures but with different degrees of nanoscale organization—more ordered in the case of cellulase and more disordered after NaOH treatment.

**FIGURE 7:**
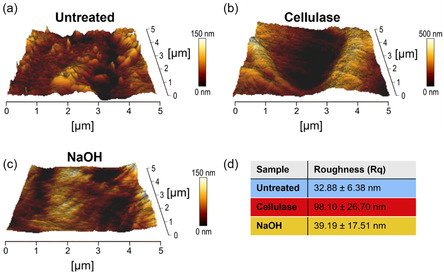
(a–c) Topographic analysis of untreated (a) and cellulase‐ (b) and NaOH‐treated (c) CNF‐based membranes evaluated by QI mode AFM (5 × 5 µm^2^, images in a 3D‐like view) after sample swelling for 30 min in PBS. (d) Table reports the surface roughness (Rq, root–mean‐square) of the membranes. Values denote mean ± standard deviation from at least five maps (5 × 5 µm^2^).

To evaluate the effect of the treatments on surface topography after swelling in aqueous environments, untreated (Figure 7a) and treated (Figure 7b,c) CNF‐based membranes were analyzed by high‐resolution force vs distance maps acquired in AFM‐QI mode, after soaking the samples in PBS. The resulting surface roughness (Rq, reported in Figure 7d) was comparable to that measured on dry samples for both untreated (32.88 ± 6.38) nm and NaOH‐treated membranes (39.19 ± 17.51) nm. In contrast, cellulase‐treated membranes exhibited a Rq value approximately 2 times higher than the dry counterpart (98.10 ± 26.70) nm. This result may be attributed to a locally heterogeneous swelling that increases surface roughness.

### 2.5 Nanomechanical Characterization of CNF‐Based Membranes

**FIGURE 8:**
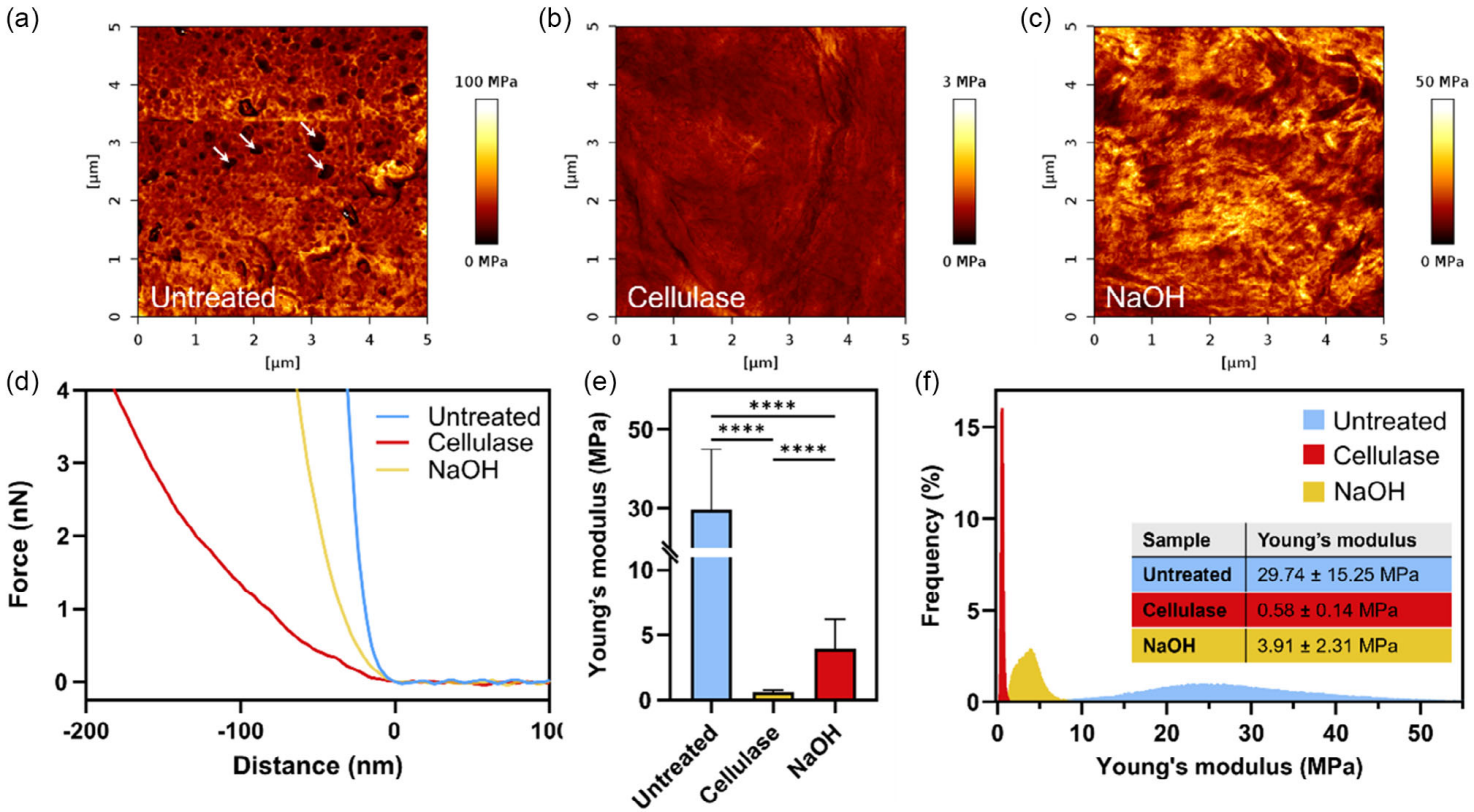
(a–c) Stiffness maps of untreated (a) and cellulase‐ (b) and NaOH‐treated (c) CNF‐based membranes, collected through force vs distance measurements in QI mode AFM (5 × 5 µm^2^, 112 × 112 pixels), with every pixel corresponding to a force vs distance measurement. (d–f) Examples of force vs distance curves obtained through QI mode AFM (d), average Young's moduli of the three analyzed samples (e), and relative frequency distribution (f) calculated from more than 25,000 data points extracted from QI mode AFM maps. The histograms in (e) and the table in (f) report Young's modulus values as mean ± standard deviation. Statistical significance: ****p < 0.0001 (one‐way ANOVA).

The nanomechanical properties of CNF‐based membranes after swelling in PBS were investigated by force–distance measurements using AFM‐QI mode. The resulting Young's modulus maps (Figure 8a–c) clearly reveal significant modifications in the surface stiffness induced by different treatments.

Although these measurements are not purely topographic, they provide valuable insights into fiber organization under wet conditions. The stiffness map of the untreated sample (Figure 8a) shows stiffer regions surrounding softer, circular areas (indicated by white arrows), resulting in an overall porous‐like appearance. This behavior is likely related to the fibrous nature of the membrane. During scanning, the AFM tip records higher Young's modulus values when in contact with the fibers, while lower stiffness values are measured in the voids between them. This morphology is more evident than in the dry sample and may therefore be accentuated by swelling.

This morphology is altered by both treatments, with stiffness maps no longer displaying pore‐like features (Figure 8b,c). These changes are accompanied by a marked modification of the mechanical properties, as summarized in Figure 8d–f. Untreated membranes exhibit a Young's modulus of (29.74 ± 15.25) MPa. The relatively high standard deviation reflects the heterogeneous structure of the wet membrane, characterized by softer pores interspersed among stiffer regions. This behavior is further confirmed by the Young's modulus frequency distribution (Figure 8f), which shows a broad range of values between 10 and 50 MPa for untreated samples. Following treatments, the Young's modulus decreases substantially to 0.58 ± 0.14 and 3.91 ± 2.31 MPa for cellulase‐ and NaOH‐treated membranes, respectively. This reduction in stiffness is accompanied by a narrower modulus distribution, indicating more uniform mechanical properties after treatment.

### 2.6 FTIR Spectroscopy of CNF‐Based Membranes

Following the morphological characterization of CNF‐based membranes and the assessment of the fibrils structural changes induced by cellulase and NaOH treatments, it was essential to investigate whether these modifications also affect the chemical structure and crystallinity of the material. Fourier‐transform infrared (FTIR) spectroscopy was therefore employed to compare pristine CNF‐based membranes with those subjected to enzymatic and chemical treatments. This analysis focused on the spectral regions characteristic of carboxymethylated CNFs, enabling the identification of possible alterations in hydrogen bonding, functional groups, and polysaccharide backbone integrity.

To this end, we first compared the carboxymethylated CNF‐based membranes with reference papers (absorbent and optical papers) to identify their distinctive peaks. Subsequently, we compared the evolution of these peaks as effect of cellulase and NaOH treatments.

**FIGURE 9:**
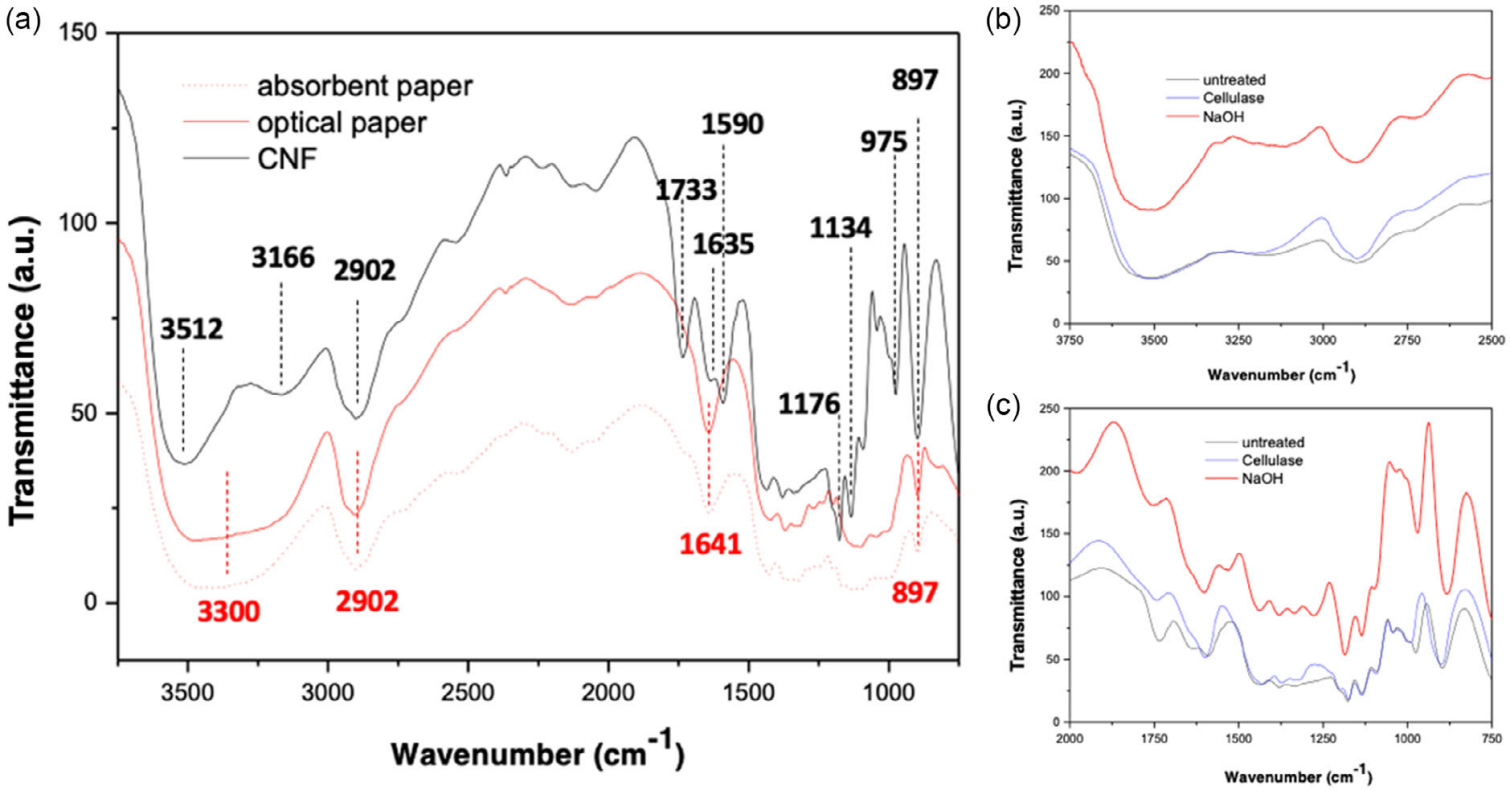
FTIR spectra of (a) carboxymethylated CNFs (black line), optical (red line) and absorbent papers (red dotted line). The main peaks are labeled. Spectra of untreated (black line) and cellulase‐treated (blue line) and NaOH‐treated (red line) carboxymethylated CNF‐based membranes in the regions of (b) 3750–2500 cm^−1^ and (c) 2000–750 cm^−1^. The reported spectra are representative of at least three samples for treatment.

Figure 9a represents the spectra of carboxymethylated CNFs in comparison with those of standard absorbent and optical papers. All samples show typical cellulose peaks, although some differences are observable. The broad peak in the spectral region 3000–3700 cm^−1^ (centered at 3300 cm^−1^) is attributed to the ‐OH stretching vibrations of hydrogen bonding interactions, due to the hydrophilicity of cellulose [39]. The peaks at 2900 cm^−1^ are related to the C–H stretching vibrations of cellulose; the absorption bands around 1640 cm^−1^ correspond to the bending modes of water molecules absorbed in the cellulose material [40]. The peaks at 897 cm^−1^ correspond to C–H rock vibrations of the β‐glycosidic bond [40].

The main differences between carboxymethylated CNFs and absorbent/optical papers are related to the presence of carboxymethylated groups and the higher purity of CNFs. Indeed, carboxymethylation introduces a new reconstruction and a strong asymmetry of the broad peak in the spectral region 3000–3700 cm^−1^, which shows for CNFs two main contributions centered at 3512 and 3166 cm^−1^, respectively. These features correspond to the stretching vibration of the ‐OH in the ‐COOH groups and could be related to inter‐ and intramolecular H‐bonding interactions [41].

Additional peaks are observed at 1733 and 1590 cm^−1^, attributable to the stretching vibrations of the ‐COOH and carboxylate (COO^‐^) groups of the carboxymethylated groups [42]. As a result of the higher purity of CNFs compared to other papers, the bands at 1176, 1134, and 975, attributable to the C–O–C and C–O stretching vibrations of the polysaccharide, appear well resolved in the CNF spectra [43].

Figures 9b–c show a comparison between FTIR spectra acquired on pristine (untreated) CNF‐based membranes and on membranes after cellulase and NaOH treatments. The regions of interest are those related to the peculiarity of the carboxymethylated CNFs structure compared to other papers, i.e., the broad peak area between 3000–3700 cm^−1^ and the region below 2000 cm^−1^ which best shows significant peaks related to both cellulose (CNFs type) and carboxymethyl groups.

From a thorough analysis of the spectra shown in Figure 9b,c, it can be concluded that no damage to the chemical bonds of cellulose or changes in peak shape or resolution are visible, which means that both treatments do not significantly alter the chemical properties of the membranes.

## 3 Discussion

In this study, we investigated carboxymethyl‐derivatized CNFs as a promising biodegradable material to develop membranes for in vitro barrier systems and thus propose it as an alternative to more conventional artificial materials such as polycarbonate, as well as to fully natural biopolymers like collagen. Despite cellulose not being a component of the mammalian ECM, CNF‐based membranes exhibit several properties that make them highly attractive for biomedical applications [19]. Their natural origin, combined with biocompatibility, biodegradability, and intrinsic optical transparency, allows these membranes to mimic certain key aspects of the BM, particularly its 3D fibrillar architecture [19, 23]. This structural similarity potentially facilitates more physiologically relevant cellular interactions compared to conventional artificial materials, which typically lack micro‐ and nanofibrillar structuring. Moreover, the environmental sustainability, cost‐effectiveness, and widespread availability of cellulose add practical advantages, especially for scalable and portable OoC systems.

Furthermore, CNF‐based membranes offer clear advantages even over pure cellulose membranes used in microfluidic BBB models, such as those reported by Duong et al. [18]. Those systems encountered notable limitations, including intrinsic autofluorescence, optical opacity, and limited porosity that impaired both imaging capabilities and direct cell–cell communication across the barrier. Our CNF‐based membranes address these issues by offering enhanced optical clarity and reduced autofluorescence, enabling high‐quality bright‐field and fluorescence microscopy—critical features for real‐time visualization and quantitative analyses in barrier models. Furthermore, the fabrication process allows for tuneable membrane thickness, providing an additional handle to tailor barrier properties according to specific experimental needs [24].

A critical aspect of any in vitro barrier system is the ability to modulate permeability to mimic physiological and pathological states [44]. We developed and validated two distinct strategies for permeability tuning: chemical treatment with NaOH and enzymatic treatment with cellulase. Both treatments significantly increased the permeability of CNF‐based membranes to small molecules such as RhodB without compromising the structural integrity or cytocompatibility of the membranes. This was demonstrated by consistent high viability of HCMEC/D3 cultured on treated membranes. Notably, the treatments could be finely controlled to avoid excessive degradation, ensuring membrane stability suitable for prolonged experiments. Moreover, the RhodB release test confirmed that small soluble compounds—such as nutrients, metabolites, or pharmacological agents—can freely diffuse without significant loss due to membrane sequestration.

Morphological and mechanical analysis revealed important insights into how these treatments influence the CNFs network. In dry state, while BF and SEM images suggested only minor surface smoothing after NaOH treatment and little visible change after cellulase exposure, AFM provided nanoscale resolution of surface features. AFM demonstrated that the original fibrous CNFs surface transforms into a granular morphology post‐treatment, with cellulase inducing the formation of compact grains composed of smaller, self‐assembled nanofibers, whereas NaOH treatment yielded more irregular rod‐like cellulose clusters. These differences likely reflect the distinct mechanisms of action: cellulase enzymatically cleaves β‐1,4 glycosidic bonds along fibrils, promoting controlled degradation, while NaOH induces swelling and partial dissolution via ionic exchange and fiber disintegration.

Mechanical analysis under wet conditions, performed by Quantitative Imaging (QI), enabled the rapid acquisition of Young's modulus maps together with high‐resolution topographic images, allowing direct correlation between surface morphological changes and stiffness variations induced by enzymatic and chemical treatments. This combined morpho‐mechanical characterization provided insight into the underlying fiber organization in physiologically relevant hydrated conditions, which are crucial for cell adhesion studies.

In the untreated samples, marked inhomogeneities were observed in the liquid state, appearing as circular pore‐like structures alternating with more irregular surface features. These pores, which likely arise from swelling between densely packed CNFs fibers, exhibited a higher apparent softness than the surrounding regions. This is consistent with the fact that the indentation was superficial and therefore not influenced by the underlying rigid substrate.

AFM image analysis relative to the control revealed no detectable morphological changes after chemical treatment, whereas enzymatic treatment led to a significant increase in surface roughness. This effect can be attributed to the localized, fiber‐oriented action of cellulase, resulting in heterogeneous surface morphology and stiffness distribution. Indeed, cellulase selectively cleaves β‐1,4‐glycosidic bonds while progressing along the fiber axis, while NaOH induces a more random partial dissolution through ionic‐exchange mechanisms.

Accordingly, samples treated with cellulase, displaying smaller nanofibers self‐assembled into compact grains in phase contrast images in dry environment, exhibited a highly inhomogeneous structure when analyzed in the wet state, consistent with the enzyme's mechanism of action. In line with the disruptive effects of both treatments, measurements in liquid showed a pronounced reduction in stiffness for both cellulase‐ and NaOH‐treated samples, reflecting substantial alterations in the CNFs nanostructure.

The measured Young's modulus in liquid falls within the range reported for wet cellulose‐based networks and AFM measurements on hydrated cellulose fibers (1–190 MPa) [45]. The observed decrease in stiffness after chemical or enzymatic treatment is advantageous, as it brings the mechanical properties of CNF‐based membranes closer to those of the native BM, which is generally reported to exhibit a Young's modulus below 5 MPa [7]. Moreover, it enables tuning of the CNFs substrate stiffness down to 0.58 MPa. This mechanical modulation is particularly relevant in tissue engineering applications, for providing the correct cell–substrate interaction or to guide cell adhesion, growth and differentiation through specific mechanical stimuli. Indeed, cell culture substrates should ideally match the mechanical properties of the native tissue they aim to replace or model in vitro. For instance, mechanical properties of substrates and matrices can strongly influence endothelial cell behavior, angiogenesis, and vasculogenesis [46], can direct human mesenchymal stem cell differentiation in the absence of soluble cues [47], or can preserve the self‐renewal of stem cells [48].

Beyond its implications in tissue engineering, the mechanical properties of the substrate are equally crucial in in vitro barrier systems. Cells forming biological barriers are highly mechanosensitive and continuously respond to the elastic properties of their microenvironment [2]. Depending on the tissue, the Young's modulus spans several orders of magnitude: approximately 1.0 kPa in adipose tissue, 0.3–0.8 MPa in vasculature, 1–20 MPa in skin, 10–40 MPa in cartilage, 50–100 MPa in tendon and ligament, and up to ∼20 GPa in bone [4]. In contrast, commonly used track‐etched membranes exhibit stiffness values in the gigapascal range (1.9–2.9 GPa), which are far from the mechanical properties of soft vascular tissues, yet they remain widely adopted for blood vessel and blood–brain barrier modeling due to their commercial availability [4]. Such mechanical mismatch can influence cell adhesion, cytoskeletal tension, tight junction organization, and ultimately barrier integrity and permeability. Therefore, the ability to tune the Young's modulus of CNF‐based membranes through controlled chemical or enzymatic treatments represents a significant advantage, enabling the development of barrier platforms with mechanical properties closer to those of native vascular tissues and thus improving the physiological relevance of in vitro models.

From a chemical perspective, FTIR analysis showed that neither treatment significantly altered the fundamental cellulose chemical bonds or crystallinity, reinforcing the conclusion that the structural framework of CNFs remains largely intact despite the fibril fragmentation observed at the microscale. This chemical stability supports the observed maintenance of membrane function and biocompatibility after treatment.

The versatility of the mortar layer integration technique further enhances the potential of CNF‐based membranes for OoC applications in PDMS chips. This approach allows reliable assembly of membranes within PDMS devices without the need for surface chemical activation, providing flexibility in device design and compatibility with various materials and experimental setups. However, a larger body of literature documents the necessity of substituting PDMS with alternative materials or developing surface‐modification strategies [9, 10] to reduce molecular absorption that can compromise, or at least influence, the outcome of biomolecular assays performed in culture media, such as drug permeability studies [49, 50, 51]. Although surface coatings are less critical in terms of fabrication methodologies, this approach is not particularly appealing for PDMS due to its strong tendency to undergo hydrophobic recovery and surface rearrangement over time, which limits the long‐term effectiveness and stability of surface modifications [52, 53]. Therefore, despite requiring the development of alternative fabrication procedures, changing the chip material represents a more robust and reliable solution, as it guarantees a stable surface chemistry.

Among the materials proposed for OoC fabrication, cyclic olefin copolymer (COC) has attracted significant attention due to its biocompatibility, chemical resistance, optical transparency, and compatibility with scalable manufacturing processes [54, 55]. Other well‐documented alternatives include polystyrene, polysulfone and glass, which also exhibit reduced molecular absorption and improved compatibility with biomolecular assays compared to PDMS [56, 57]. Leveraging a wide range of materials and microfabrication techniques is crucial for advancing barrier‐on‐chip technologies that require precise control over physical and biochemical microenvironments.

An additional advantage of CNF‐based membranes lies in the possibility of exploiting their intrinsic biodegradability to integrate them into more environmentally friendly LoC and OoC platforms, using fabrication technologies compatible with polymers such as polylactide, poly(DL‐lactic‐co‐glycolide), and other bio‐derived and biodegradable materials. This aspect is particularly relevant given that many disposable microfluidic devices significantly contribute to plastic waste, with the impact in biomedical laboratories estimated to reach approximately 1000 kg per year [58].

Taken together, these results establish CNF‐based membranes as a highly promising platform for developing advanced in vitro barrier models. Their combination of tuneable permeability, optical clarity, biocompatibility, and ease of integration addresses multiple limitations of existing materials. The ability to mimic essential structural and functional features of the BM paves the way for more physiologically relevant studies, including tissue barrier modeling, drug transport, and disease modeling under both static and dynamic flow conditions.

## 4 Conclusions

Carboxymethylated CNF‐based membranes represent an alternative to porous artificial membranes and overcome the optical and structural limitations of conventional cellulose membranes, offering high transparency, low autofluorescence, and tuneable thickness. Their biocompatibility was demonstrated through successful endothelial cell culture, and their permeability and stiffness could be modulated via mild NaOH or cellulase treatments without compromising chemical stability or cell viability. The release profiles also confirmed that both untreated and treated membranes had a similar ability to release small molecules at regular 24‐h intervals.

NaOH treatment induced controlled ionic swelling and surface smoothing, while cellulase promoted partial nanofibrillar degradation, both approaches enabling predictable tuning of membrane permeability. Morphological, mechanical, chemical, and functional characterizations confirmed that these modifications preserved the fundamental integrity of CNFs while providing flexibility for application‐specific optimization.

The combination of optical clarity, chemical stability, biocompatibility, and tuneable transport properties positions CNF‐based membranes as promising candidates for advanced in vitro barrier models, particularly in microfluidic organ‐on‐chip systems. Future studies will investigate the performance of tuneable CNF‐based membranes under dynamic flow conditions, in coculture configurations, and in long‐term biological applications, both within PDMS devices and within chips fabricated from bio‐derived and fully biodegradable polymers. In this context, the ultimate goal of this latter strategy—namely, integrating CNF‐based membranes into chips composed of biodegradable materials—is to advance manufacturing approaches that reduce the overall environmental impact of LoC and OoC platforms.

## 5 Methods

### 5.1 Membrane Fabrication

Nanocellulose membranes were prepared from carboxymethylated CNFs, prepared according to a procedure described by Wågberg et al. [24], and were supplied by RISE Bioeconomy, Stockholm, Sweden, as a 2wt% hydrogel. The CNFs had a degree of modification corresponding to 600 µmolg^−1^ carboxylates. Membranes were prepared from the CNFs by first diluting the CNFs hydrogel with deionized water to a 0.2 wt% colloidal dispersion using an Ultra Turrax high‐intensity mixer for 10 min followed by centrifugation for 1 h to remove larger aggregates. The membranes were subsequently formed by dead end filtration of the diluted CNFs in a vacuum funnel equipped with a PVDF filter (Durapore) with a pore size of 0.45 µm. The filter cake consisting of the collected CNFs were completely dried in a Rapid Köthen sheet dryer at reduced pressure and 93°C for 15 min. The dried membranes were finally cured for 1 h at 150°C.

### 5.2 Dual‐Chamber Device Fabrication

CNF‐based membranes were placed between layers of PDMS (Sylgard 184 Dowsil) in devices featuring a dual‐chamber design. The PDMS was prepared by combining PDMS base and curing agent at a 10:1 weight ratio, followed by degassing to eliminate any trapped air. Subsequently, PDMS slabs were created by pouring the degassed mixture into a Petri dish and curing it at 140°C for 15 min. The device design was the same as a previously published work [59]. Two square PDMS slabs (1.5 cm × 1.5 cm) were then punched to create milli‐chambers with a diameter from 3 to 8 mm. CNF‐based membranes was flattened using a hydraulic press (15 kN, 50°C lower press/100°C upper press, 5 min) and placed between the punched PDMS slabs. The devices were sealed using a mortar layer polymerization process (100°C for 10 min) involving a PDMS solution first deposited onto a glass slab by spin‐coating (500 rpm × 60 s, followed by 2500 rpm × 20 s) and then transferred onto the inner side of the PDMS slabs. For all experiments, the dual‐chamber devices were placed on a glass coverslip to seal the bottom chamber after filling it with specific solutions according to the experiments.

### 5.3 Membrane Treatments, Permeability Assay, and Release Test

CNF‐based membranes, situated between the layers of dual‐chamber devices, underwent distinct treatments aimed at modifying the membrane permeability. One treatment involved the addition of a 2 wt% NaOH solution to the top compartment of the device, left for 2 min at room temperature, followed by three rinses in distilled water. Another treatment utilized cellulase from *Trichoderma* sp. at a concentration of 5 mg/mL in PBS with pH = 7 (Corning), incubated in the top compartment for 3 h at 37°C, followed by three rinses in distilled water.

**FIGURE 10:**
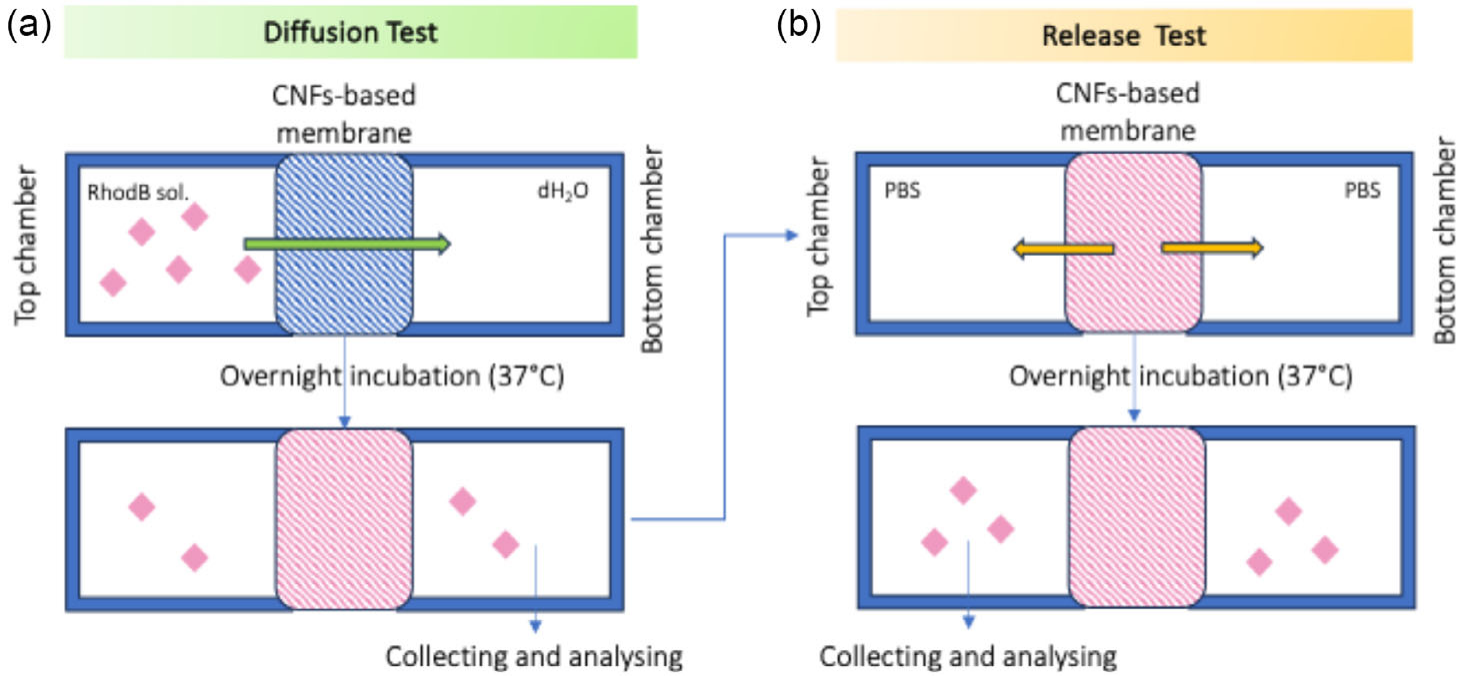
Diffusion and Release Tests. Scheme of (a) diffusion of RhodB trough CNF‐based membranes and (b) following release of the RhodB retained in the membranes during the diffusion test.

The permeability of CNF‐based membranes (untreated, cellulase‐treated, and NaOH‐treated) was evaluated using RhodB (1 mg/mL in distilled water) as a fluorescent tracer. The RhodB solution was introduced into the upper chamber (donor compartment), while the lower chamber (acceptor compartment) was filled with distilled water. Devices were incubated overnight at 37°C to allow tracer diffusion (Figure 10a).

After 20 h, the solution from the acceptor chamber was collected, and RhodB absorbance was measured using a microplate reader (CLARIOstar Plus, BMG LABTECH). Absorbance values were converted into concentrations using a pre‐established RhodB calibration curve. The apparent permeability (*P*_app_) was calculated according to the equation reported by Motallebnejad et al. [60]

$$P_{\mathrm{app}}=C_{f}\cdot V/C_{i}\cdot A\cdot t$$

where C_*f*_ is the RhodB concentration in the acceptor compartment after incubation (determined from the calibration curve), V is the volume of the acceptor chamber (60 µL), *C*_*i*_ is the initial RhodB concentration in the donor compartment (1 mg/mL), *A* is the membrane area between chambers (50.24 mm^2^), and *t* is the incubation time (20 h).

Following the permeability assay, the same CNF‐based membrane samples (untreated, cellulase‐treated, and NaOH‐treated) were subjected to sequential release experiments. Each device was incubated at 37°C, filling both chambers with PBS for 24 h. At the end of the incubation, the PBS solution from the top chamber was collected, and RhodB absorbance was measured to determine the amount of dye released from the membranes (Figure 10b). The procedure was repeated three consecutive times, filling the top chamber with fresh PBS for each cycle. Absorbance values, proportional to RhodB concentration, were plotted as a function of cumulative incubation time, calculated as 24 h for the first cycle, 48 h for the second, and 72 h for the third.

### 5.4 Membrane Characterization

For the microstructural analysis of the cellulose membrane, the uncoated specimens were studied by a scanning electron microscope (Zeiss Sigma 300 VP) to obtain secondary electron images and by an optical microscope (Zeiss LSM 980) at 10× magnification to obtain bright‐field images.

AFM images in air were acquired by a Park instrument (XE‐100) in noncontact mode, using silicon rectangular probes with a tip radius of 10 nm. For each sample, different areas were analyzed to ensure the reproducibility of the results. Image analysis was performed by the XEI software. The root mean square roughness (Rq) values were calculated on an area of 5 µm x 5 µm.

Force–distance measurements and surface topography roughness analyses of wet samples were carried out using a newly developed Bio‐Hybrid System named LEoPard (LEP). This platform integrates a JPK Nanowizard V Atomic Force Microscope (Bruker, Germany) with a fluorescence and bright‐field microscope (Axio Observer, Zeiss, Germany). The entire setup was housed within a closed environmental enclosure and mounted on an anti‐vibration table to reduce external mechanical disturbances [61, 62]. The measurements were conducted in liquid at room temperature, after soaking membranes in PBS for 30 min, using a ScanAsyst‐Fluid cantilever (Bruker) equipped with a triangular pyramidal tip (nominal radius: 20 nm; spring constant: 0.7 N/m). Topographic and Young's modulus maps were obtained using an acquisition mode named “Quantitative Imaging (QI™) advanced mode.” This force spectroscopy imaging technique generates spatial maps of the sample and provides quantitative information on parameters including height, adhesion, and mechanical properties. At each pixel, a complete force–distance curve was recorded by indenting the sample surface until a predefined force threshold was reached. A setpoint force of 5 nN and a z‐range of 500 nm were applied.

At least five maps (5 × 5 µm^2^, 112 × 112 pixels) were acquired per sample. Each force–distance curve was fitted pixel by pixel using the Hertz contact model, as implemented in the JPK analysis software (Bruker BioAFM Handbook v. 8.1).

Surface roughness (Rq, root mean square) was determined under the same acquisition settings by evaluating the height data obtained in QI mode. For each sample, at least five randomly selected, nonoverlapping images (5 × 5 µm^2^) were acquired and used for roughness analysis.

Absorbent and optical paper as well as untreated and NaOH/cellulase‐treated CNF‐based membranes were studied by FT‐IR. The analyses were performed with a Jasco FT/IR 6300 spectrophotometer using an ATR accessory. Infrared spectroscopy measurements were carried out in the 4000–400 cm^−1^ spectral range.

### 5.5 Cell Culture

Human cerebral microvascular endothelial cells (HCMEC/D3) (Cederlane Laboratories, Canada, Catalog No. CLU512, RRID:CVCL_U985) were cultured in Ham's F12 (Sigma–Aldrich/Merk) supplemented with 5% fetal bovin serum (Sigma–Aldrich/Merk), 5% L‐glutamine (Sigma–Aldrich/Merk), 0.25% penicillin (10,000 IU/mL), streptomycin (10,000 μg/mL) (Sigma–Aldrich/Merk), 1 μg/mL hydrocortisone 21‐hemisuccinate sodium salt (Sigma–Aldrich/Merk), 50 μg/mL L‐ascorbic acid (Sigma–Aldrich/Merk), 0.75 U/mL heparin (Sigma–Aldrich/Merk), and 1 ng/mL human basic fibroblast growth factor (Sigma–Aldrich/Merk).

### 5.6 Cell Growth on CNF‐Based Membranes

Before seeding the cells, the dual‐chamber devices were sterilized using UV exposure for 30 min and the degradation treatments (with NaOH or cellulase) followed. Additionally, a preconditioning treatment (with PBS for 3 h at 37°C) was needed to equilibrate the NaOH‐treated and untreated membranes. Then, a fibronectin coating was applied to the top side of the interposed CNF‐based membrane by incubation with 50 µg/mL fibronectin in PBS solution for 45 min at room temperature, followed by two washes with PBS. HCMEC/D3 cells were then cultured at a density of 7 × 10^5^ cells/cm^2^ on the fibronectin‐coated cellulose membrane, with the cell medium being changed after 24 h. After 48 h of culture, cell growth and viability were evaluated using optical live imaging and fluorescence imaging (EVOS M7000 Thermofisher). In particular, live and dead assays (Live/Dead Kit, Invitrogen) were conducted to confirm the cytocompatibility of the treated membranes.

### 5.7 Statistical Analysis

All experiments were performed at least three times or repeated in three batches of independent experiments. Data were presented as the mean ± standard deviations. Statistical significance was assessed using one‐way ANOVA; **** indicates *p* < 0.0001.

## Author Contributions

**Vita Guarino**: conceptualization (lead), data curation (lead), investigation (lead), methodology (lead), writing – original draft (lead), writing – review and editing (lead). **Johan Erlandsson**: investigation (equal), methodology (equal), writing – review and editing (equal). **Elisa De Luca**: methodology (equal). **Elisabetta** **P** **errone**: methodology (equal). **Alessandra Zizzari**: methodology (supporting). **Monica Bianco**: methodology (supporting). **Albert Portone**: investigation (supporting), methodology (supporting). **Stefano Leporatti**: investigation (supporting), methodology (supporting). **Lars Wågberg**: investigation (equal), methodology (equal). **Giuseppe Gigli**: funding acquisition (lead), methodology (supporting). **Lorenzo Moroni**: conceptualization (supporting), data curation (supporting), supervision (supporting), writing – review and editing (supporting). **Valentina Arima**: conceptualization (equal), data curation (equal), investigation (supporting), methodology (supporting), supervision (lead), writing – review and editing (equal).

## Declarations

During the preparation of this work, the authors used ChatGPT in order to refine their English. After using this tool/service, the authors reviewed and edited the content as needed and takes full responsibility for the content of the publication.

## Funding

This study was supported by Regione Puglia (“Tecnopolo per la medicina di precisione” ‐TecnoMed Puglia —Regione Puglia: DGR n.2117 del 21/11/2018, CUP: B84I1800054000; "Biotecnologia, bioinformatica e sviluppo farmaceutico” per la creazione di un hub delle scienze di vita, piano operativo salute (fsc 2014–2020), traiettoria 4, azione 4.1—cod. t4‐an‐01—Regione Puglia, and CUP: f83c22001560003).

## Conflicts of Interest

The authors declare no conflicts of interest.

## Data Availability

The data that support the findings of this study are available from the corresponding author upon reasonable request.
